# The cross-interaction between global and age-comparative self-rated health on depressive symptoms–considering both the individual and combined effects

**DOI:** 10.1186/s12888-016-1098-9

**Published:** 2016-12-05

**Authors:** Jaeyong Shin, Eun-Cheol Park, Sang Gyu Lee, Young Choi, Jae-Hyun Kim, Tae Hyun Kim

**Affiliations:** 1 Department of Preventive Medicine, Yonsei University, College of Medicine, Seoul, South Korea; 2 Institute of Health Services Research, Yonsei University, College of Medicine, Seoul, South Korea; 3 Department of Hospital Administration, Graduate School of Public Health, Yonsei University, 50 Yonsei-ro, Seodaemun-gu, Seoul, 210-752 South Korea; 4 Department of Public Health, Graduate School, Yonsei University, Seoul, South Korea; 5 Department of Preventive Medicine and Public Health, Ajou University School of Medicine, Suwon, South Korea; 6 Institute on Aging, Ajou University Medical Center, Suwon, South Korea

**Keywords:** Self-rated health, Combined, Age comparative, Depression, Old aged

## Abstract

**Background:**

Numerous studies suggesting the relation between self-rated health (SRH) and depression have been reported using different measures. Therefore, we attempted to determine the difference in a depressive scale based on the different ways of measuring health between global SRH (SRH-global) and age-comparative SRH (SRH-age). Then, the combined effect of SRH-global and SRH-age on depressive symptoms was further investigated.

**Methods:**

Data from the Korean Longitudinal Study of Ageing (KLoSA) from 2008 to 2012 were analyzed. We divided the SRH-global and SRH-age into three levels—high, middle, and low—and combined each into nine new categories (SRH-combi). The Center for Epidemiologic Studies Depression Scale-10 Korean edition was used as the dependent variable.

**Results:**

A total of 8621 participant were enrolled at baseline. Individuals with lower SRHs-age compared to SRH-global tended to be more vulnerable to depressive symptoms. Low SRH-global with low (*b* = 0.654, *p* < 0.001) and middle SRH-age (*b* = 0.210, *p* = 0.003) showed association with higher CESD scores. Participants with high SRH-global × low SRH-age also had higher scores (*b* = 0.536, *p* < 0.001) compared to the “middle SRH-global × middle SRH-age” reference group. In contrast, among the middle (*b* = −0.696, *p* < 0.001) and high SRH-global (*b* = −0.545, *p* < 0.001) groups, participants with superior SRH-age had statistically lower CESD scores than the reference group.

**Conclusions:**

Although a sole general SRH has historically been widely used, it has been suggested that use of both general and age-comparative SRH would be more powerful and easy when we consider analyzing depression in old age.

**Electronic supplementary material:**

The online version of this article (doi:10.1186/s12888-016-1098-9) contains supplementary material, which is available to authorized users.

## Background

Major depressive disorder (MDD) contributes the significant burden of diseases in developed countries, and there will be further increases [1–3]. According to the Korean Statistical Informational Service (KOSIS, 2011), approximately 27.6% of the general population in Korea suffers from mental disorders during a lifetime [4]. While it is more prevalent than men, 12.0 and 9.1% were observed to have anxiety disorder and MDD, respectively, among women during their lifetimes.

In addition, the Republic of Korea has become a rapidly aging society. As a result, geriatric depression has also emerged as a major social issue. It is widespread and affects at least one in six patients treated in general medical practice and an even higher percentage in hospitals and nursing homes. Depression later in life has serious consequences, including distress among patients and caregivers, which is amplified by disability associated with medical and cognitive disorders of later life, increased health care costs, and increased mortality related to suicide and medical illness. In fact, in 2011, the age-standardized suicide mortality rate in Korea was 33.3 per 100,000 individuals, the highest among all OECD countries [5].

Thus, it is important to identify the determinants associated with depressive symptoms as early as possible. In fact, numerous studies suggesting the relation between self-rated health (SRH) and depression have been reported [2, 6–8]. SRH is able to measure one’s perception of one’s general health status. This has been widely used and recognized as a validated indicator of health in a variety of populations. It also allows for comparisons across different conditions and populations [9–12].

The questions for measuring SRH could be classified according to three main categories. The first is a non-comparative SRH, which is usually measured by asking respondents whether they would rate their health as excellent, good, fair, poor, or very poor. The next is an age-comparative SRH measured by asking respondents whether they would rate their health status as better, the same, or worse if compared to that of other people their age. The last is a time-comparative SRH, in which respondents are asked to rate their health compared to how it was at a given time in the past. [13] The three different SRH measurements seem to represent parallel assessments of subjective health. However, there is a possibility of difference among the measurements. For example, people tend to overestimate their health in relation to others with increasing age [11, 13]. A recent study reported significantly positive linear trends between age-comparative SRH and physical health problems, such as respiratory diseases, musculoskeletal diseases, any active chronic diseases, functional disability, depressive symptoms, taking medication regularly, and admission to hospital last year [14]. However, those who rated their time-comparative SRH as “normal” had the smallest odds ratios in all of the physical health problems mentioned above than those who rated it as “better” or “worse”. Thus, it is necessary to compare the differences in depression using a Center for Epidemiologic Studies Depression Scale (CESD) scale based on the different ways of measuring SRH. Because studies regarding the combined effect of global and age-comparative SRH on depressive symptoms in Korea are rare, it will be valuable to investigate the differences among SRHs and the association between combined SRH and depression.

## Methods

### Study population

We used data from the Korean Longitudinal Study of Ageing (KLoSA) from the second panel survey in 2008 to the fourth in 2012. A basic survey for KLoSA has been conducted every even-numbered year starting in 2006, mainly using the same survey categories regarding social, financial, and health. The population of KLoSA includes, in principle, all adults aged 45 and over. Many other surveys of elderly people in other countries only include the population aged 50 and over. In contrast, Korea experienced a financial crisis in the late 1990s, and career changes in the population of middle-aged individuals in their late 40s became an important social issue. Therefore, KLoSA decided to extend the population to include those aged between 45 and 49. The KLoSA used the Computer Assisted Personal Interviewing (CAPI) survey method. Because age-comparative SRH was not included in the first survey conducted in 2006, we defined the starting point as the second wave in 2008.

### Ethics statement

The institutional review board from the Graduate School of Public Health, Yonsei University, approved this research (IRB approval No. 2-1040939-AB-N-01-2016-149). Since we used the national public opened data with de-identification and designed retrospective cross-sectional study, we did not seek informed consent for participation. However, KLoSA initially explained the aim of survey and collected informed consents from participants at baseline.

### Measurement on depressive symptoms

The CESD was created in 1977 by Laurie Radloff [15] and revised in 2004 by William Eaton and others [16]. The CESD has been the workhorse of depression epidemiology since its first use in the Community Mental Health Assessment Surveys in the 1970s [17, 18] and is used in the National Health and Nutrition Examination Surveys [19]. It has survived transition to the telephone as well as a self-administered version and is usable with typically undercounted populations such as the elderly and the economically disadvantaged. The scale is well known and remains one of the most widely used instruments in the field of psychiatric epidemiology [20–22]. We used the CESD-10 Korean edition for measuring depressive symptoms defined by the American Psychiatric Association’ Diagnostic and Statistical Manual (DSM-IV).

### Socio-demographic factors

These factors include age, marital status, living area, education, and economic situation. Age group was carefully classified according to four categories: 54 or below, 55 to 64, 65 to 74, and 75 or above. Living area was divided into three levels: rural area, small to medium city, and metropolitan. Educational level was classified according to four levels from elementary school to college or over. The household heads provided their annual household income level. We then divided the income level into four categories based on the quartile results: Low, Low-Middle, Middle-High, High.

### Health-related factors

These factors included whether they performed regular exercise, the number of chronic diseases, cancer history, the proportion of health expenditure in household income, type of medical security system, and whether they joined private health insurance. The number of chronic diseases was divided into four levels: none, one, two, three or more. Excessive health expenditure was defined as the proportion of health expenditures in household income and classified according to four levels from 5 % or below to 20 % or over.

We also included the type of medical security system and whether they joined private health insurance to adjust the effect of security for health on depression. In Korea, the medical security system is classified as national health insurance (NHI) or medical aid. People can qualify for medical aid if their single-family household income is < $600 per month; otherwise, they have mandatory NHI. Those who have NHI based on employment pay a monthly insurance premium according to their annual salary, and people who are self-employed pay for their premium based on the value of their property.

There are two types of private health insurance in Korea [23, 24]. The first is fixed benefit insurance, which pays a fixed amount defined in accordance with the PHI contract. Another is indemnity health insurance, which fully covers services uninsured by the NHI program and out-of-pocket payments for services covered by the NHI program. According to the national statistics [25], 76.8% of household had any kind of private health insurance in 2011.

### Measurement on SRH

In KLoSA, SRH was measured using the two following questions.

I. “How would you rate your general health status?” Reply alternatives were Excellent, Quite good, Neither good nor poor, Quite poor, and Poor (referred to subsequently as SRH-global).
II. “Now I’m going to ask you about life satisfaction. Please answer how satisfied you are with the following compared to people of your own age. How satisfied are you with your health?” The answer was measured by a continuous variable from 0 to 100 by units of ten. In other words, Zero meant absolutely dissatisfied and 100 meant absolutely satisfied. To make a comparable study design, we classified this according to five categories as Excellent (90 to 100), Quite good (70 to 80), Neither good nor poor (40 to 60), Quite poor (20 to 30), and Poor (0 to 10) (referred to subsequently as SRH-age).
III. To measure the combined effect between SRH-global and SRH-age, we re-categorized both health status variables as follows: the health status of participants was defined as High (who answer Excellent and Quite good), Middle (Neither good nor poor), or Low (Quite poor and Poor). We then made another health status variable for the combined analysis (referred to subsequently as SRH-combined). (Additional file 1: Figure S1)

### Statistical analysis

We evaluated both the separate effects of SRH-global and SRH-age and the combined effect of both variables. For analysis of combined effect, we selected the “middle SRH-global × middle SRH-age” group as reference. Differences in CESD-mean by each variable were tested using a *t*-test and ANOVA. Associations between CESD and the variables included in three different health status variables (SRH-global, SRH-age, and SRH-combined) and other covariates (socio-demographic and health-related) were initially analyzed with product-moment correlation. As a second step, multiple linear regression analyses (PROC GENMOD; SAS procedure) were used separately for the different SRHs with CESD as the dependent variable, and the variables were included in the factors as independent variables. To compare the goodness of fit among three different SRHs, Quasi-Akaike Information Criterion (QIC) was also applied. In general, the lower value was relatively better than the others. Statistical analyses were performed using SAS, version 9.3 (SAS Institute Inc., Cary, NC, US). Significant differences are indicated according to the following: * *P* < 0.05 and ** *P* < 0.001.

## Results

### General characteristics of participants

Table 1 summarizes the general characteristics of the population. Among 8688 participants of KLoSA in 2008, 67 (0.8%) were excluded due to lack of information. Thus, a total of 8621 participants were initially enrolled at baseline. Among them, 3734 men and 4887 women were included in this study. There were 3998 participants (46.4%) aged 65 or over, and 4603 (53.4%) had one or more chronic diseases such as hypertension, diabetes, hypercholesterolemia, or osteoarthritis. Five hundred and fifteen enrolled participants (6.0%) were covered by the medical aid program while the remaining participants were covered by national medical insurance; 2742 participants (31.8%) had private health insurance at baseline. The mean CESD score at baseline was 3.77 with a standard deviation (SD) of 2.96. Except for gender, there were statistical differences in all covariates. Thus, we put all of these independent variables together for analysis of the CESD scores using multivariate analysis.

**Table 1:** General characteristics and CESD 10 among subjects at the baseline

	General characteristics		CESD 10 score	*p*-value
	N	%	Mean ± SD	
Gender				0.387
Male	3734	43.3	3.78 ± 2.93	
Female	4887	56.7	3.75 ± 2.97	
Age				<0.001
−54	2241	26.0	3.48 ± 2.84	
55–64	2382	27.6	3.63 ± 2.90	
65–74	2485	28.8	3.92 ± 3.00	
75−	1513	17.6	4.16 ± 3.09	
Marital status				<0.001
Single	1954	22.7	4.94 ± 2.99	
Married	6667	77.3	3.42 ± 2.85	
Region				<0.001
Metropolitan	3738	43.4	3.50 ± 2.90	
Small to Medium city	2763	32.0	3.78 ± 3.00	
Rural area	2120	24.6	4.21 ± 2.93	
Regular exercise				<0.001
No	5555	64.4	4.13 ± 3.02	
Yes	3066	35.6	3.11 ± 2.72	
Employment		0.0		<0.001
Employed	3616	41.9	2.92 ± 2.63	
Unemployed	5005	58.1	4.37 ± 3.03	
Educational level				<0.001
Elementary	3942	45.7	4.60 ± 2.99	
Middle	1474	17.1	3.61 ± 2.89	
High	2291	26.6	2.92 ± 2.68	<0.001
College or above	914	10.6	2.55 ± 2.44	
Income level		0.0		
Lowest	2170	25.2	5.02 ± 3.00	
Lower	2428	28.2	3.85 ± 2.91	
Higher	2146	24.9	3.19 ± 2.78	<0.001
Highest	1877	21.8	2.86 ± 2.63	
Number of chronic diseases				
None	4018	46.6	3.05 ± 2.72	
One	2602	30.2	3.93 ± 2.94	
Two	1329	15.4	4.70 ± 2.95	<0.001
Three or more	672	7.8	5.62 ± 2.97	
Cancer history				
Yes	8388	97.3	2.95 ± 0.03	
No	233	2.7	3.01 ± 0.20	
Excessive health expenditure				<0.001
< 5.0 %	5166	59.9	3.38 ± 2.84	
5.0–9.9 %	1372	15.9	3.86 ± 2.94	
10.0–19.9 %	950	11.0	4.26 ± 3.05	<0.001
20.0 % −	1133	13.1	5.01 ± 2.99	
Type of medical guarantee		0.0		
Insurance	8106	94.0	3.65 ± 2.93	
Medical aid	515	6.0	5.52 ± 2.87	
Co-coverage from private health insurance				<0.001
Yes	2742	31.8	2.82 ± 2.58	
No	5879	68.2	4.20 ± 3.02	
Total	8621	100.0	3.77 ± 2.96	

### Distribution of health status variables (SRHs) among participants

According to the SRH-global, there were 2537 participants (29.4%) with ratings of Low, 3119 (36.2%) with ratings of Middle, and 2965 (34.4%) with ratings of High at baseline (Table 2). The distribution of SRH-age was as follows: 1725 participants (20.0%) with ratings of Low, 3695 (42.9%) with ratings of Middle, and 3201 (37.1%) with ratings of High in 2008. The differences in distribution between two SRH variables were small. The distributions of SRH-combined categories are summarized in Table 2. The number of reference categories with the middle SRH-global × middle SRH-age was 1700 (19.7%) at baseline. Interestingly, the largest category was High SRH-global × High SRH-age with 1912 participants (22.0%).

**Table 2:** The distribution of health status variables regarding SRH by year and CESD 10

			2008		2010				2012			
	N	%	CESD 10 score		N	%	CESD 10 score		N	%	CESD 10 score	
			Mean SD	*p*-value			Mean SD	*p*-value			Mean SD	*p*-value
SRH-global				<0.001				<0.001				<0.001
Low	2537	29.4	4.10 ± 3.06		2372	30.0	4.00 ± 3.10		2226	29.8	5.22 ± 3.07	
Middle	3119	36.2	3.85 ± 2.93		2945	37.3	3.67 ± 2.95		3093	41.4	3.17 ± 2.72	
High	2965	34.4	3.39 ± 2.85		2583	32.7	3.57 ± 2.97		2153	28.8	2.64 ± 2.47	
SRH-age				<0.001				<0.001				<0.001
Low	1725	20.0	4.25 ± 2.99		1525	19.3	4.18 ± 3.04		1340	17.9	5.67 ± 2.87	
Middle	3695	42.9	3.94 ± 2.94		3399	43.0	3.78 ± 2.97		3292	44.1	3.87 ± 2.91	
High	3201	37.1	3.30 ± 2.89		2976	37.7	3.46 ± 2.99		2840	38.0	2.38 ± 2.40	
SRH-Combi (SRH-global and SRH-age)				<0.001				<0.001				<0.001
LL [Low-Low]	1218	14.1	4.23 ± 3.04		1095	13.9	4.17 ± 3.09		977	13.1	6.16 ± 2.77	
LM [Low-Middle]	1088	12.6	4.06 ± 3.05		1061	13.4	3.89 ± 3.09		1025	13.7	4.66 ± 3.10	
LH [Low-High]	231	2.7	3.55 ± 3.11		216	2.7	3.70 ± 3.16		224	3.0	3.76 ± 3.00	
ML [Middle-Low]	351	4.1	4.46 ± 2.89		287	3.6	4.06 ± 3.00		260	3.5	4.31 ± 2.82	
MM [Middle-Middle]	1700	19.7	4.06 ± 2.92		1596	20.2	3.79 ± 2.91		1611	21.6	3.57 ± 2.75	
MH [Middle-High]	1068	12.4	3.32 ± 2.88		1062	13.4	3.38 ± 2.97		1222	16.4	2.40 ± 2.47	
HL [High-Low]	156	1.8	3.90 ± 2.77		143	1.8	4.50 ± 2.77		103	1.4	4.58 ± 2.59	
HM [High-Middle]	907	10.5	3.58 ± 2.83		742	9.4	3.60 ± 2.93		656	8.8	3.39 ± 2.75	
HH [High-High]	1902	22.1	3.26 ± 2.86		1698	21.5	3.48 ± 2.98		1394	18.7	2.14 ± 2.14	
Total	8621	100.0	3.77 ± 2.96		7900	100.0	3.74 ± 3.00		7472	100.0	3.62 ± 2.96	

### Multivariate analysis using SRH-global and SRH-age

Before the multivariate analysis using SRH-combined, we first performed multivariate analyses using SRH-global and SRH-age with three levels (Table 3). According to the results, it appears that CESD-10 scores were increased with statistically significant difference when both the SRH-global and SRH-age were low (SRH-global; *b* = 0.51, *p* < 0.001, SRH-age; *b* = 0.52, *p* < 0.001). In contrast, the estimates for CESD-10 scores were decreased when both SRH variables were high, although there was statistical difference only in SRH-age (*b* = −0.59, *p* < 0.001) and not SRH-global (*b* = −0.07, *p* = 0.130).

**Table 3:** Multivariate analysis among all subjects, without the interaction between SRH-global and SRH-age

	CESD on SRH-global			CESD on SRH-age		
	beta	SE	*p*-value	beta	SE	*p*-value
SRH						
Low	0.51	0.05	<.001	0.52	0.06	<.001
Middle	ref			ref		
High	−0.07	0.05	0.130	−0.59	0.05	<.001

The other covariates including gender, age, marital status, living area, regular exercise, emplyment, educational level, income level, number of chronic diseases, cancer history, excessive health expenditure, type of medical guarantee and co-coverage from private health insurance were adjusted

**Fig. 1:**
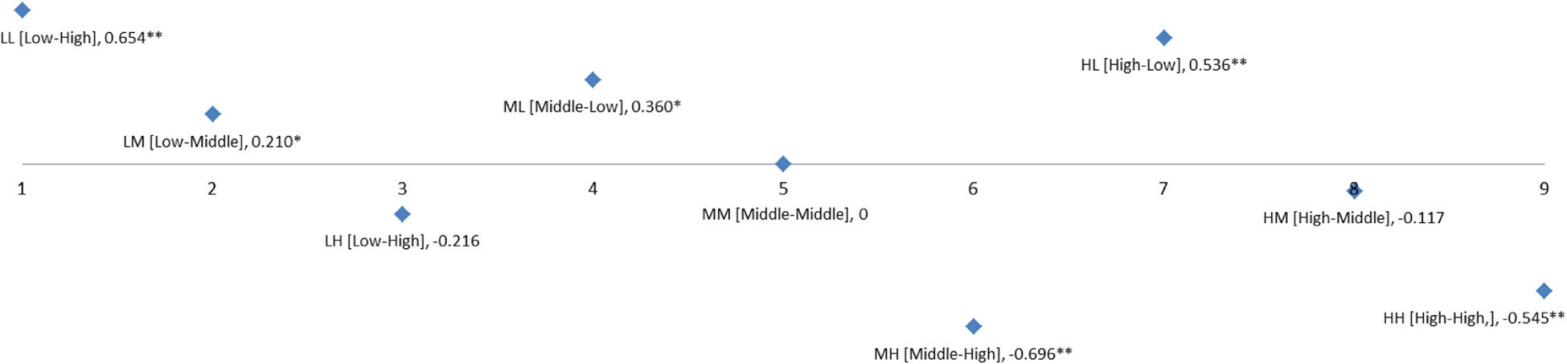
CESD by each nine SRH-combined categories. CESD means the center for epidemiologic studies depression scale, which is a screening test for depression and depressive disorder. In all SRH-global levels including low, middle, and high, the low SRH-age showed a statistically increase in CESD. In contrast, in middle and high SRH-global levels, relatively high SRH-age was statistically associated with the increase in CESD. *P < 0.05, **P < 0.001

To determine the combined effect of SRH-global and SRH-age on depressive symptoms, we used another health status variable, SRH-combined (Table 4). According to the result, the low SRH-global with low (*b* = 0.654, *p* < 0.001) and middle (*b* = 0.210, *p* = 0.003) SRH-age showed association with the increased CESD score with statistically significant difference. Interestingly, participants with high SRH-global × low SRH-age also had a higher CESD score (*b* = 0.536, *p* < 0.001) compared to the reference group “middle SRH-global × middle SRH-age.” In contrast, participants whose SRH-age was superior to their SRH-global had significantly lower CESD than the reference group among the middle (*b* = −0.696, *p* < 0.001) and high SRH-global (*b* = −0.545, *p* < 0.001) groups Fig. 1.

**Table 4:** Multivariate analysis among all subjects, with the combined effect (SRH-combined) between general SRH (SRH-global) and age-comparative SRH (SRH-age)

	CESD		
	beta	SE	*p*-value
Combined effect beetween current health status and expected health status in the aged			
LL [Low-Low]	0.654	0.075	<.0001
LM [Low-Middle]	0.210	0.071	0.003
LH [Low-High]	−0.216	0.128	0.093
ML [Middle-Low]	0.360	0.106	0.001
MM [Middle-Middle]	ref		
MH [Middle-High]	−0.696	0.066	<.0001
HL [High-Low]	0.536	0.143	<.0001
HM [High-Middle]	−0.117	0.074	0.115
HH [High-High]	−0.545	0.064	<.0001
Year			
2008	ref		
2010	−0.046	0.036	0.204
2012	−0.158	0.037	<.0001
Sex			
Male	0.131	0.044	0.003
Female	ref		
Age			
−54	ref		
55–64	−0.092	0.057	0.108
65–74	−0.051	0.070	0.471
75−	0.203	0.083	0.015
Marital status			
Married	ref		
Single	0.119	0.055	0.029
Region			
Metropolitan	−0.369	0.063	<.0001
Small to Medium city	−0.092	0.066	0.162
Rural area	ref		
Regular exercise			
No	0.149	0.044	<.0001
Yes	ref		
Employment			
Employed	−0.240	0.048	<.0001
Unemployed	ref		
Educational level			
Elementary	ref		
Middle	0.045	0.060	0.453
High	−0.078	0.058	0.179
College or above	−0.230	0.078	0.003
Income level			
Lowest	ref		
Lower	−0.007	0.059	0.903
Higher	−0.079	0.064	0.219
Highest	−0.084	0.072	0.245
Number of chronic diseases			
None	ref		
One	−0.035	0.048	0.467
Two	0.040	0.062	0.519
Three or more	0.088	0.080	0.276
Cancer history			
Yes	ref		
No	−0.030	0.112	0.788
Excessive health expenditure			
< 5.0 %	ref		
5.0–9.9 %	0.091	0.055	0.096
10.0–19.9 %	0.122	0.066	0.066
20.0 %−	0.288	0.066	<.0001
Type of medical guarantee			
Insurance	−0.448	0.095	<.0001
Medical aid	ref		
Co-coverage from private health insurance			
Yes	0.019	0.048	0.701
No	ref		

Regarding the other covariates, singles (*b* = 0.119, *p* = 0.029) had a slightly increased CESD score compared to married participants. In terms of living area, participants living in metropolitan areas (*b* = −0.369, *p* < 0.001) had significantly lower CESD scores than those in rural areas. In educational level, participants with college education or over (*b* = −0.230, *p* = 0.003) only showed decreased CESD scores compared to those with elementary school education. The employees (*b* = −0.230, *p* = 0.003) among the participants demonstrated significantly negative association with CESD.

Compared to those with medical aid, participants with medical insurance (*b* = −0.448, *p* < 0.001) showed association with low CESD scores, and participants in households in which medical expense/total income was 20 % or above presented significantly increased CESD scores (*b* = 0.288, *p* = 0.003) compared to the reference participants with household expenses for medical cost less than five percent of total house income.

We also performed subgroup analysis by income group for both SRH-global and SRH-age (Additional file 2: Figure S2). We observed increasing CESD scores according to the increased income groups among low SRH-global and SRH-age. In contrast, there was no definite statistical tendency to decrease CESD scores in both higher SRHs. However, the magnitudes of CESD scores between low and high SRHs were both the greatest in the highest quartile income group.

### Subgroup analysis by gender using SRH-combined

Male participants rating low SRH-age matched with all kinds of SRH-global (low; *b* = 0.825, *p* < 0.001, middle; *b* = 0.472, *p* = 0.006, high; *b* = 0.696, *p* = 0.001) had high CESD scores compared to the reference “middle SRH-global × middle SRH-age” group (Additional file 3: Figure S3). Similarly, women with low SRH-global × low SRH-age (*b* = 0.553, *p* < 0.001) and middle SRH-global × low SRH-age (*b* = 0.286, *p* = 0.037) indicated that association with higher CESD scores. However, there was no statistical difference in women in the “high SRH-global × low SRH-age” group regardless of the positive estimate (*b* = 0.377, *p* = 0.064).

### Subgroup analysis by quartile income groups using SRH-combined

**Fig. 2:**
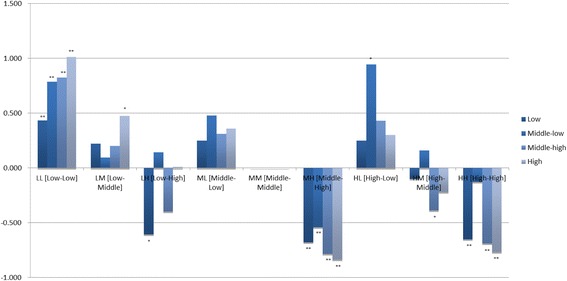
CESD by each nine SRH-combined categories, by income quartile groups from low to high consequence. CESD means the center for epidemiologic studies depression scale, which is a screening test for depression and depressive disorder. Among the low SRH-global * low SRH-age group [LL], all estimates of CESD had positive value compared to the reference group with statistical difference and the beta estimates were increased as the income was increased (low; beta = 0.433, *p* < 0.001, middle-low; beta = 0.788, *p* < 0.001, middle-high; beta = 0.823, *p* < 0.001, high; beta = 1.011, *p* <0.001). Interestingly, only the middle-low quartile group in the middle SRH-global * the low SRH-age ([ML], *b* = 0.478, *p* = 0.013) and the high SRH-global * the low SRH age ([HL], *b* = 0.946, *p* = 0.001) categories showed statistically differences. *P < 0.05, **P < 0.001

In the low “SRH-global × low SRH-age” category [LL], estimates for CESD scores in all quartile income groups had positive value compared to the reference [MM], and the beta estimates increased as the income increased (low; beta = 0.433, *p* < 0.001, middle-low; beta = 0.788, *p* < 0.001, middle-high; beta = 0.823, *p* < 0.001, high; beta = 1.011, *p* < 0.001). In the “middle SRH-global × the high SRH-age” group [MH], all quartile groups had lower CESD scores compared to the reference group (low; beta = −0.680, *p* < 0.001, middle-low; beta = −0.542, *p* < 0.001, middle-high; beta = −0.785, *p* < 0.001, high; beta = −0.837, *p* < 0.001). Interestingly, only the middle-low quartile group in the “middle SRH-global × the low SRH-age” ([ML], *b* = 0.478, *p* = 0.013) and “high SRH-global × the low SRH age” ([HL], *b* = 0.946, *p* = 0.001) categories showed statistical differences (Fig. 2).

## Discussion

According to the results, there are statistically different variances among the estimates for CESD scores within the same SRH-global levels among different SRH-age levels. Even though the SRH-global level was high, the estimate for CESD was statistically increased in low SRH-age. Conversely, when the SRH-global level was low, there was no significant difference from the reference “middle SRH-global × middle SRH-age” group. Other covariates of marital status, regular exercise, employment, educational level, and excessive health expenditure presented similar results to those of previous studies.

Review of previous papers regarding depressive symptoms was difficult using SRH-age and not SRH-global [26, 27]. As we performed the same methods as those used in the previous studies using SRH-global and SRH-age alone, the results from both health status variables showed similar trends to the CESD. The QIC values, which are indicators of goodness of fit, were almost the same between the two SRH variables (SRH-global; QIC = 23,911.3, QICu = 23,905/SRH-age; QIC = 23,911.2, QICu = 23,905). In this sense, it is necessary to measure the effect of the combined SRH on depressive symptoms in another way. To make the interpretation easier, we reduced the five levels of the original SRH-global and SRH-age into three and made another nine SRH-combined categories by three methods. Despite the fact that we could not verify statistical significance, we observed the reduction of power in SRH-combined (SRH-combined; QIC = 23,917.5, QICu = 23,911).

Based on the subgroup analysis by gender, it appeared that men had a higher magnitude by the change of SRH-combined categories. In other words, men might be more sensitive to self-related health. However, in another study from Hong Kong, men were more likely to report “better” and less likely to report “worse” SRH than were women [14]. Thus, in order to determine this difference between genders, further investigation is needed.

In addition, according to the subgroup analysis by the quartile income groups, the highest income group was the most sensitive to changes in SRH-combined. Similar or opposite trends were also observed in other SRH-related research [28, 29]. Burstrom and Fredlund investigated the relationship between SRH and subsequent mortality across individual’s socioeconomic classes [28]. Similarly, the association between less than good SRH and mortality rate appeared stronger in higher than in lower socioeconomic individuals. However, according to another study in the United Kingdom [29], there was no interaction between SRH and socioeconomic classes in their effect on risk of deaths. Although there are reasonable hypotheses to explain why the effect of SRH on health outcomes might differ across socioeconomic classes in both directions, it is not clear why SRH would have a weaker effect in high socioeconomic individuals in some countries or stronger in others. However, in our study, we cautiously suggest that depressive symptoms might be largely associated with economic situation among lower socioeconomic groups, especially in Korea [30, 31]. Thus, SRH in the higher socioeconomic group might have greater association with depressive symptoms compared to SRH in the lower socioeconomic group.

When we looked inside the categories in which SRH-age was inferior to SRH-global, such as middle SRH-global × low SRH-age [ML], high SRH-global × low SRH-age [HL], and high SRH-global × middle SRH-age [HM], the middle-low income group was the most vulnerable to the change of SRH-combined. In fact, it is well known that income changes and the time dimension of income are important for SRH [32–34]. SRH responds to decreases in absolute income and lowered rank position in the income distribution to a greater extent than it does to income gains over time. However, it is very interesting that the group most vulnerable to depressive symptoms by the change of SRH-combined is the second lowest group, not the lowest one. We suggested that Medical Aid, a special medical security system for the poor in Korea, covered a large portion of these vulnerable participants in the lowest quartile groups while it could not in the second lowest group.

SRH is one of the most frequent measurements, assessing health perceptions in many epidemiological studies. Several previous studies suggested that even though other important covariates, including physical, socio-demographic, and psycho-social health characteristics, were adjusted, the individual’s self-assessment for his or her own global health could be a powerful indicator for further morbidity and mortality [35–38]. Several hypotheses have explained these results as follows. First, SRH might be associated with some illness that could not be detected by medical science. Another theory is that SRH is able to reflect one’s lifestyle behaviors as well as psycho-social and socio-demographic conditions known to be related to health outcomes [39]. Finally, excessive anxiety regarding health has been proved to have important association with poor SRH [40–42]. In summary, SRH could be an indicator for the instability of the masked physical and mental sickness in one’s general health.

In this context, SRH could offer an easy and efficient way to identify patients at risk for poor long-term depression outcomes [2]. According to one previous study from Australia [2], cross-sectional analysis of baseline data showed that participants reporting poor or fair SRH had greater odds of chronic illness, MDD, and lower socioeconomic status than those reporting good to excellent SRH. For participants rating their health as poor to fair compared with those rating it good to excellent, risk ratios of MDD were 2.10 (95% CI, 1.60–2.76), 2.38 (95% CI, 1.77–3.20), 2.22 (95% CI, 1.70–2.89), 1.73 (95% CI, 1.30–2.28), and 2.15 (95% CI, 1.59–2.90) at 1, 2, 3, 4, and 5 years, respectively, after accounting for missing data us19pt?>after the adjustment for other covariates (Additional file 4: Table S1). In another study [6], a decrease in depressive symptoms was associated with increased odds for having better SRH (OR, 1.15, 95% CI; 1.04–1.27). Interestingly, it can be expanded to all age groups even though the target population was patients with type 2 diabetes [7]. To put all the things together, the SRH was a strong direct predictor of depressive symptoms and patients’ functional health.

In this study, SRH was measured as follows: Participants were asked to estimate their SRH on a scale ranging from 0 (“very poor”) to 10 (“very good”). Although this scoring system was different from that used in the two previous studies, it was the same method to our measurement of SRH-age. Therefore, when we put all these results together, it is clear that SRH could clearly reflect depressive symptoms or be useful and efficient indicators for the depressive disorder.

A cross-sectional survey of a nationally representative sample of Israeli persons aged 45 years or older showed that individuals aged 65 years or older were more likely to give a more favorable rating of their health when asked to compare themselves with people of the same age and sex than when the rating was made without a comparison instruction [43]. Heckhausen insisted that self-enhancement is a more important motive in later life because of the need to stabilize the self-amidst increasing difficulties in controlling events in life such as a major health problem [44]. As a result, the effect of self-enhancement on SRH should be greater for older than for younger people. In line with this thinking, we hypothesize that SRH is a stronger function of social comparison in the physical domain for older than for younger adults and that such comparisons serve as a buffer for older people against the threat to SRH due to increasing physical problems. Thus, greater consideration of the SRH-combined, which is the summary of SRH-global and SRH-age, as an important factor is needed when using the SRH in analysis of depression among older people.

There are several limitations in this study.

- First, we could not expand this result to all age groups. Since this KLoSA panel was designed to determine the characteristics of families with older persons aged 65 or over, the participants here could be left-truncated at the baseline.
- Second, we could not evaluate whether there is a real effect of SRH-age changes with age. As previous studies conducted in other countries mentioned that SRH-age had greater association with older than with younger people, it was necessary to evaluate this using other national data in Korea. However, it was impossible that KLoSA is the only Korean national survey using SRH-age until now.
- Third, SRH could differ from one culture to another, even if the questions are the same [45, 46]. For example, self-enhancement may be a less salient motive for Asians [47, 48]. Additionally, SRH might have different values across ethnicity. Among African Americans, SRH does not have predictability for long-term predictive power to mortality compared to the Caucasians in America [49]. Thus, it should be carefully interpreted when this result is applied in other sociocultural backgrounds.
- Fourth, we could not generalize the association of SRH and various health outcomes. Although SRH is a good indicator for predicting mortality rate, mortality is not as same as depressive symptom in this research. Thus, we clearly mention that it should be differentiated when you compare the results to other health outcomes like comorbidity or mortality.
- Finally, SRH-age is not exactly the same as in other studies. Other studies [26, 27] measured the comparative SRH as follows: “How would you assess your general health condition compared to persons of your own age?” with the alternatives “Better,” “Worse,” or “Similar.” However, our indicator also measured age-comparative satisfaction with health reflecting overall health and well-being as the same as others, and we operationally defined SRH-age. Regardless of the similarity, you carefully interpreted the outcome compared to others in different studies.

## Conclusions

Although global SRH is a well-known indicator for estimating depressive disorder, it is suggested that use of both general SRH and age-comparative SRH would be more powerful when considering analyzing depression. In conclusion, individuals with lower SRHs-age compared to SRH-global tend to be more vulnerable to depressive symptoms.

## Additional files

Additional file 1:
**Figure S1.** Combined variables between global self-rated health (SRH-global) and age-comparative self-rated health (SRH-age). (JPG 56 kb)
Additional file 2:
**Figure S2.** CESD by SRH-global and SRH-age separately, according to the quartile income group. SRH means self-rated health. SRH-global is general self rated health, while SRH-age is age-comparative self –rated health. The vertical axis means the estimates of CESD and the horizontal one is four income categories from low to high group. * *p* < 0.05 ***p* < 0.001. (JPG 80 kb)
Additional file 3:
**Figure S3.** Values of estimates for CES-D by each nine SRH-combined categories by gender. SRH means self-rated health. SRH-global is general self rated health, while SRH-age is age-comparative self–rated health. Among men, subjects with low SRH-age and all kinds of SRH-global levels (low; *b* = 0.825, *p* < 0.001, middle; *b* = 0.472, *p* = 0.006, high; *b* = 0.696, *p* = 0.001) had higher CESD compared to the reference middle SRH-global * middle SRH-age group. Similarly, women with low SRH-global * low SRH-age (*b* = 0.553, *p* < 0.001) and middle SRH-global * low SRH-age (*b* = 0.286, *p* = 0.037) showed association with higher CESD. However, there was no statistical difference in women with high SRH-global * low SRH-age group although the estimate was positive (*b* = 0.377, *p* = 0.064). (DOCX 18 kb)
Additional file 4:
**Table S1.** The association between covariates and SRHs. SRH means self-rated health. SRH-global is general self rated health, while SRH-age is age-comparative self–rated health. Chi-square tests were performed for statistical tests. (21 kb)

## References

[1] Mathers C, Fat DM, Boerma JT, World Health Organization (2008). The Global Burden of Disease: 2004 Update.

[2] Ambresin G, Chondros P, Dowrick C, Herrman H, Gunn JM (2014). Self-rated health and long-term prognosis of depression. Ann Fam Med.

[3] Bloom DE, Cafiero ET, Jané-Llopis E (2011). The global economic burden of non-communicable diseases.

[4] Statistics Korea: Life time prevalence of psychological disorders in Korea 2013. http://kosis.kr/statisticsList/statisticsList_01List.jsp?vwcd=MT_ZTITLE&parentId=D#SubCont. 12 March 2015.

[5] Organisation for Economic Co-operation and Development (2013). Health at a glance 2013 : OECD indicators.

[6] Han B, Jylha M (2006). Improvement in depressive symptoms and changes in self-rated health among community-dwelling disabled older adults. Aging Ment Health.

[7] Boehme S, Geiser C, Renneberg B (2014). Functional and self-rated health mediate the association between physical indicators of diabetes and depressive symptoms. BMC Fam Pract.

[8] Jahn DR, Cukrowicz KC (2012). Self-rated health as a moderator of the relation between functional impairment and depressive symptoms in older adults. Aging Ment Health.

[9] Idler EL, Benyamini Y (1997). Self-rated health and mortality: a review of twenty-seven community studies. J Health Soc Behav.

[10] Ostrove JM, Adler NE, Kuppermann M, Washington AE (2000). Objective and subjective assessments of socioeconomic status and their relationship to self-rated health in an ethnically diverse sample of pregnant women. Health Psychol.

[11] Vuorisalmi M, Lintonen T, Jylha M (2006). Comparative vs global self-rated health: associations with age and functional ability. Aging Clin Exp Res.

[12] Adler NE, Epel ES, Castellazzo G, Ickovics JR (2000). Relationship of subjective and objective social status with psychological and physiological functioning: preliminary data in healthy white women. Health Psychol.

[13] Eriksson I, Unden AL, Elofsson S (2001). Self-rated health. Comparisons between three different measures. Results from a population study. Int J Epidemiol.

[14] Li ZB, Lam TH, Ho SY, Chan WM, Ho KS, Li MP, Leung GM, Fielding R (2006). Age- versus time-comparative self-rated health in Hong Kong Chinese older adults. Int J Geriatr Psychiatry.

[15] Radloff LS (1977). The CES-D scale: a self-report depression scale for research in the general population. Appl Psychol Meas.

[16] Eaton WW CM, Smith C, Tien A, Ybarra M, Maruish ME (2004). Center for epidemiologic studies depression scale: review and revision (CESD and CESD-R). The use of psychological testing for treatment planning and outcomes assessment.

[17] Comstock GW, Helsing KJ (1976). Symptoms of depression in two communities. Psychol Med.

[18] Radloff LS, Locke BZ (1986). Community mental health assessment survey and the CES-D Scale.

[19] Eaton WW, Kessler LG (1981). Rates of symptoms of depression in a national sample. Am J Epidemiol.

[20] Murphy JM (2002). Symptom scales and diagnostic schedules in adult psychiatry.

[21] Naughton MJ, Wiklund I (1993). A critical review of dimension-specific measures of health-related quality of life in cross-cultural research. Qual Life Res.

[22] Snaith P (1993). What do depression rating scales measure?. Br J Psychiatry.

[23] Shin J (2012). Private health insurance in South Korea: an international comparison. Health Policy.

[24] Choi Y, Kim JH, Yoo KB, Cho KH, Choi JW, Lee TH, Kim W, Park EC (2015). The effect of cost-sharing in private health insurance on the utilization of health care services between private insurance purchasers and non-purchasers: a study of the Korean health panel survey (2008–2012). BMC Health Serv Res.

[25] National Health Insurance Cooperation. A report on the Korea Health Panel Survey (2008 ~ 2011). Seoul: Korea Institute for Health and Social Affairs, Korea National Health Insurance Corporation. 2013.

[26] Waller G, Janlert U, Hamberg K, Forssen A (2016). What does age-comparative self-rated health measure? A cross-sectional study from the Northern Sweden MONICA Project. Scand J Public Health.

[27] Dai Y, Zhang CY, Zhang BQ, Li Z, Jiang C, Huang HL (2016). Social support and the self-rated health of older people: A comparative study in Tainan Taiwan and Fuzhou Fujian province. Medicine (Baltimore).

[28] Burstrom B, Fredlund P (2001). Self rated health: Is it as good a predictor of subsequent mortality among adults in lower as well as in higher social classes?. J Epidemiol Community Health.

[29] McFadden E, Luben R, Bingham S, Wareham N, Kinmonth AL, Khaw KT (2009). Does the association between self-rated health and mortality vary by social class?. Soc Sci Med.

[30] Park JN, Han MA, Park J, Ryu SY (2016). Prevalence of Depressive Symptoms and Related Factors in Korean Employees: The Third Korean Working Conditions Survey (2011). Int J Environ Res Public Health.

[31] Shin J, Choi JW, Jang SI, Choi Y, Lee SG, Ihm TH, Park EC (2015). The temporal association of excessive health expenditure with suicidal ideation among primary income earners: a cross-sectional design using the Korean Welfare Panel Survey (KoWePS). BMJ Open.

[32] Miething A, Aberg Yngwe M (2014). Stability and variability in income position over time: exploring their role in self-rated health in Swedish survey data. BMC Public Health.

[33] Kondo N, Sembajwe G, Kawachi I, van Dam RM, Subramanian SV, Yamagata Z (2009). Income inequality, mortality, and self rated health: meta-analysis of multilevel studies. Br Med J.

[34] Benzeval M, Judge K (2001). Income and health: the time dimension. Soc Sci Med.

[35] Kaplan GA, Camacho T (1983). Perceived health and mortality: a nine-year follow-up of the human population laboratory cohort. Am J Epidemiol.

[36] Idler EL, Angel RJ (1990). Self-rated health and mortality in the NHANES-I Epidemiologic Follow-up Study. Am J Public Health.

[37] Appels A, Bosma H, Grabauskas V, Gostautas A, Sturmans F (1996). Self-rated health and mortality in a Lithuanian and a Dutch population. Soc Sci Med.

[38] Mossey JM, Shapiro E (1982). Self-rated health: a predictor of mortality among the elderly. Am J Public Health.

[39] Idler EL, Kasl S (1991). Health perceptions and survival: do global evaluations of health status really predict mortality?. J Gerontol.

[40] Barsky AJ, Cleary PD, Klerman GL (1992). Determinants of perceived health status of medical outpatients. Soc Sci Med.

[41] Fylkesnes K, Forde OH (1991). The Tromso Study: predictors of self-evaluated health--has society adopted the expanded health concept?. Soc Sci Med.

[42] Fylkesnes K, Forde OH (1992). Determinants and dimensions involved in self-evaluation of health. Soc Sci Med.

[43] Baron-Epel O, Kaplan G (2001). General subjective health status or age-related subjective health status: does it make a difference?. Soc Sci Med.

[44] Heckhausen J (1999). Developmental regulation in adulthood: agenormative and sociostructural constraints as adaptive challenges.

[45] Cheng ST, Fung H, Chan A (2007). Maintaining self-rated health through social comparison in old age. J Gerontol Ser B Psychol Sci Soc Sci.

[46] Kim G, DeCoster J, Chiriboga DA, Jang Y, Allen RS, Parmelee P (2011). Associations between self-rated mental health and psychiatric disorders among older adults: do racial/ethnic differences exist?. Am J Geriatr Psychiatry.

[47] Kitayama S, Markus HR, Matsumoto H, Norasakkunkit V (1997). Individual and collective processes in the construction of the self: self-enhancement in the United States and self-criticism in Japan. J Pers Soc Psychol.

[48] Ross M, Heine SJ, Wilson AE, Sugimori S (2005). Cross-cultural discrepancies in self-appraisals. Personal Soc Psychol Bull.

[49] Assari S, Lankarani MM, Burgard S (2016). Black-white difference in long-term predictive power of self-rated health on all-cause mortality in United States. Ann Epidemiol.

