# Supplementary figures and images for: The cross-interaction between global and age-comparative self-rated health on depressive symptoms–considering both the individual and combined effects
Source: BMC Psychiatry. 2016 Dec 5;16:433. doi: 10.1186/s12888-016-1098-9 (PMC5139095; doi:10.1186/s12888-016-1098-9)

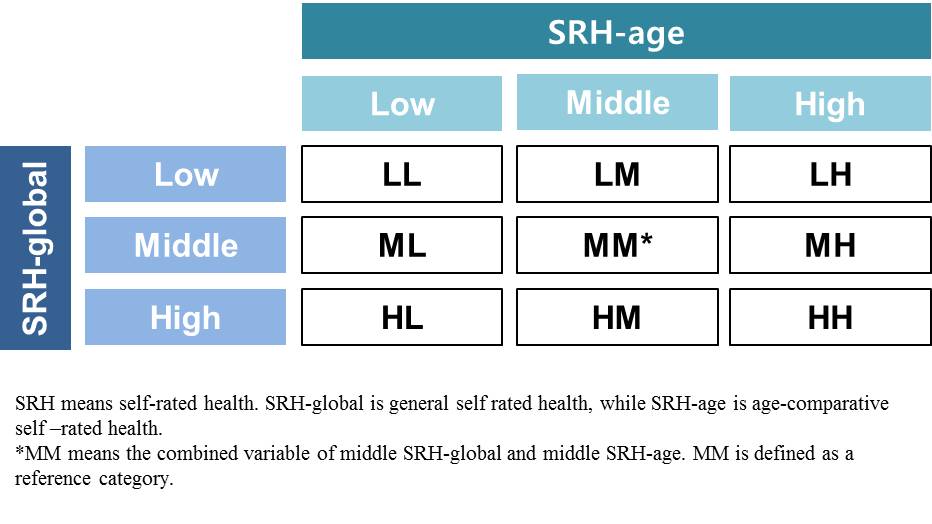

Supplement: Additional file 1: — Figure S1. Combined variables between global self-rated health (SRH-global) and age-comparative self-rated health (SRH-age). (JPG 56 kb) [file 12888_2016_1098_MOESM1_ESM.jpg]

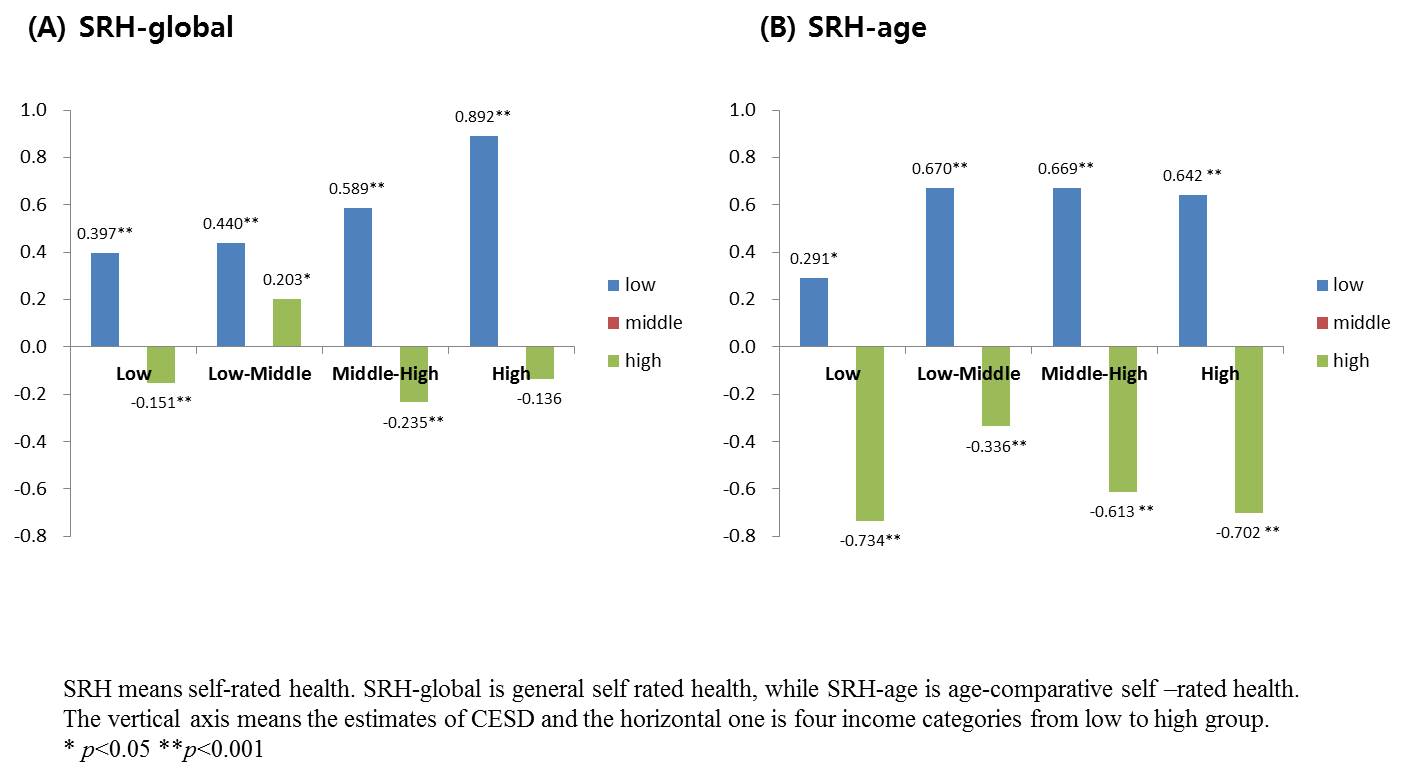

Supplement: Additional file 2: — Figure S2. CESD by SRH-global and SRH-age separately, according to the quartile income group. SRH means self-rated health. SRH-global is general self rated health, while SRH-age is age-comparative self –rated health. The vertical axis means the estimates of CESD and the horizontal one is four income categories from low to high group. * p < 0.05 **p < 0.001. (JPG 80 kb) [file 12888_2016_1098_MOESM2_ESM.jpg]
