# Supplementary material for: The cross-interaction between global and age-comparative self-rated health on depressive symptoms–considering both the individual and combined effects
Source: BMC Psychiatry. 2016 Dec 5;16:433. doi: 10.1186/s12888-016-1098-9 (PMC5139095; doi:10.1186/s12888-016-1098-9)
Supplement: Additional file 3: — Figure S3. Values of estimates for CES-D by each nine SRH-combined categories by gender. SRH means self-rated health. SRH-global is general self rated health, while SRH-age is age-comparative self–rated health. Among men, subjects with low SRH-age and all kinds of SRH-global levels (low; b = 0.825, p < 0.001, middle; b = 0.472, p = 0.006, high; b = 0.696, p = 0.001) had higher CESD compared to the reference middle SRH-global * middle SRH-age group. Similarly, women with low SRH-global * low SRH-age (b = 0.553, p < 0.001) and middle SRH-global * low SRH-age (b = 0.286, p = 0.037) showed association with higher CESD. However, there was no statistical difference in women with high SRH-global * low SRH-age group although the estimate was positive (b = 0.377, p = 0.064). (DOCX 18 kb) [file 12888_2016_1098_MOESM3_ESM.docx]

**Supplementary figure 3.** Values of estimates for CES-D by each nine SRH-combined categories, by gender

Among men, subjects with low SRH-age and all kinds of SRH-global levels (low; b=0.825, p<0.001, middle; b=0.472, p=0.006, high; b=0.696, p=0.001) had higher CESD compared to the reference middle SRH-global * middle SRH-age group. Similarly, women with low SRH-global * low SRH-age (b=0.553, p<0.001) and middle SRH-global * low SRH-age (b=0.286, p=0.037) showed association with higher CESD. However, there was no statistical difference in women with high SRH-global * low SRH-age group although the estimate was positive (b=0.377, p=0.064).
